# Comprehensive Bioinformatics Analysis Reveals the Potential Role of the hsa_circ_0001081/miR-26b-5p Axis in Regulating COL15A1 and TRIB3 within Hypoxia-Induced miRNA/mRNA Networks in Glioblastoma Cells

**DOI:** 10.3390/biomedicines12102236

**Published:** 2024-10-01

**Authors:** Bartosz Lenda, Marta Żebrowska-Nawrocka, Ewa Balcerczak

**Affiliations:** Department of Pharmaceutical Biochemistry and Molecular Diagnostics, Medical University of Lodz, Muszynskiego 1, 90-151 Lodz, Poland; marta.zebrowska@umed.lodz.pl (M.Ż.-N.); ewa.balcerczak@umed.lodz.pl (E.B.)

**Keywords:** glioblastoma, ceRNA, U-87 MG cells, GBM molecular subtype, GBM mesenchymal subtype, collagen, bioinformatics

## Abstract

**Background/Objectives:** The intrinsic molecular heterogeneity of glioblastoma (GBM) is one of the main reasons for its resistance to conventional treatment. Mesenchymal GBM niches are associated with hypoxic signatures and a negative influence on patients’ prognosis. To date, competing endogenous RNA (ceRNA) networks have been shown to have a broad impact on the progression of various cancers. In this study, we decided to construct hypoxia-specific microRNA/ messengerRNA (miRNA/mRNA) networks with a putative circular RNA (circRNA) regulatory component using available bioinformatics tools. **Methods:** For ceRNA network construction, we combined publicly available data deposited in the Gene Expression Omnibus (GEO) and interaction pairs obtained from miRTarBase and circBank; a differential expression analysis of GBM cells was performed with limma and deseq2. For the gene ontology (GO) enrichment analysis, we utilized clusterProfiler; GBM molecular subtype analysis was performed in the Glioma Bio Discovery Portal (Glioma-BioDP). **Results:** We observed that miR-26b-5p, generally considered a tumor suppressor, was upregulated under hypoxic conditions in U-87 MG cells. Moreover, miR-26b-5p could potentially inhibit *TRIB3*, a gene associated with tumor proliferation. Protein-protein interaction (PPI) network and GO enrichment analyses identified a hypoxia-specific subcluster enriched in collagen-associated terms, with six genes highly expressed in the mesenchymal glioma group. This subcluster included hsa_circ_0001081/miR-26b-5p-affected *COL15A1*, a gene downregulated in hypoxic U-87 MG cells yet highly expressed in the mesenchymal GBM subtype. **Conclusions:** The interplay between miR-26b-5p, *COL15A1*, and *TRIB3* suggests a complex regulatory mechanism that may influence the extracellular matrix composition and the mesenchymal transformation in GBM. However, the precise impact of the hsa_circ_0001081/miR-26b-5p axis on collagen-associated processes in hypoxia-induced GBM cells remains unclear and warrants further investigation.

## 1. Introduction

Although glioblastoma (GBM), an isocitrate dehydrogenase-wildtype (IDH-wt) World Health Organization (WHO) grade 4 tumor, is one of the best molecularly characterized human malignancies, it still presents a significant clinical problem due to poor overall patient survival and limited treatment options. Those include surgical resection, followed by radiotherapy and chemotherapy with an alkylating agent such as temozolomide [1,2,3]. GBM is characterized by an invasive phenotype and high levels of both intra- and intertumoral heterogeneity. This creates an immense need for the development of personalized approaches with the aim of targeting not only different types of gliomas but individual tumors as well [4,5]. Thus, it is still important to deepen our understanding of the GBM molecular landscape with an emphasis on different cellular niches, the tumor microenvironment (TME), and various physiological processes that, by mutual interactions, are entangled to form a complex clinical image of GBM.

It is widely known that hypoxic states are feature characteristics of the GBM microenvironment [5]. In epithelial tumors, hypoxia can induce the epithelial-to-mesenchymal transition (EMT), which increases cancer invasiveness [6,7]. In GBM, a tumor of glial origin, similar molecular cascades with analogous changes in cell behavior can also be observed, being recently referred to as the notion of the so-called proneural-to-mesenchymal transition [8,9,10]. Additionally, the GBM mesenchymal phenotype is commonly known to be associated with the glioma hypoxic signature and tumors with worse prognosis [11,12,13].

According to the ceRNA hypothesis, some coding and non-coding transcripts can compete for miRNA by acting like molecular sponges [14]. This may exert a significant regulatory effect on various signalling pathways, including the wingless-related integration site/β-catenin (Wnt/β-catenin), nuclear factor kappa-light-chain-enhancer of activated B cells (NF-κB), and the phosphatidylinositol 3-kinase/protein kinase B (PI3K/AKT) [15,16,17]. Among long non-coding RNA (lncRNA), circRNAs are characterized by their high stability in tissues and body fluids [18]. To date, circRNAs have been found to influence the pathogenesis of various human malignancies, including hepatocellular cancer, breast cancer, and glioma [19,20,21]. Regarding brain tumors, they can be especially valuable as potential liquid biopsy biomarkers [22].

**Figure 1:**
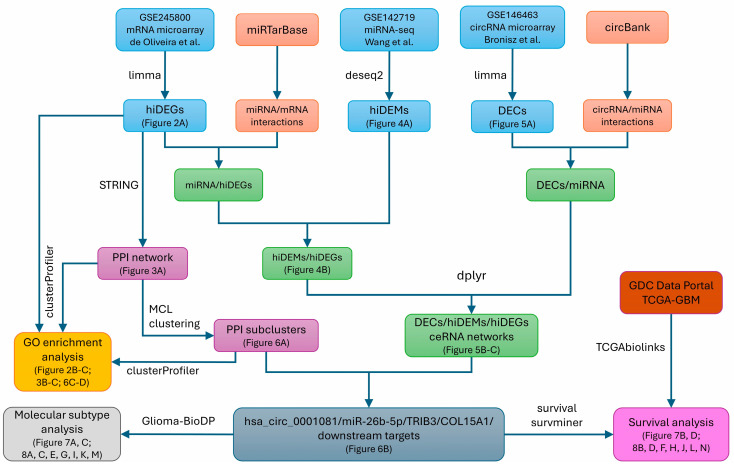
General workflow of the bioinformatics analysis. By using limma and deseq2, we conducted differential expression (DE) analyses of three publicly available datasets deposited in the Gene Expression Omnibus (GEO). Both mRNA and miRNA data consisted of U-87 MG cells treated with hypoxia and normoxia, while circRNA data consisted of GBM patient-derived cells and neural progenitor cells as a control group. By intersecting the results of DE analyses with corresponding interaction pairs from miRTarBase and circBank, we constructed putative ceRNA networks. Note that we concurrently constructed two types of networks according to the ceRNA hypothesis (this is omitted in Figure 1 to keep it more concise); this consisted of upDECs/down-hiDEMs/up-hiDEGs and a second one that consisted of down-hiDECs/up-hiDEMs/downDEGs. For all significantly expressed hypoxia-induced genes (hiDEGs; both up- and downregulated), we performed GO enrichment analysis and constructed a PPI network in Cytoscape; the PPI network was further subjected to MCL clustering, and selected clusters were subsequently analyzed with GO enrichment terms. Next, by analyzing different PPI subclusters in the context of the obtained ceRNA and GO enrichment results, we selected the hsa_circ_0001081/miR-26b-5p/*TRIB3*/*COL15A1* axis and its downstream targets for further investigation. To do so, we analyzed the protein-coding components of this axis by molecular subtype and survival analyses using data from Giloma-BioDP and the GDC Data Portal, respectively. For more detailed information about each step of the workflow, see the Materials and Methods section. For utilized R code and generated results, see DOI 10.5281/zenodo.13770443. Figure numbers corresponding to particular steps of the workflow are in brackets. GBM—glioblastoma; hiDEGs—hypoxia-induced genes; hiDEMs—hypoxia-induced miRNA; DECs—hypoxia-induced circRNA; up—upregulated; down—downregulated; PPI—protein–protein interaction; GO—gene ontology; ceRNA—competing endogenous RNA; GDC—Genomic Data Commons; TCGA—The Cancer Genome Atlas; Glioma-BioDP—Glioma Bio Discovery Portal; MCL—Markov Cluster Algorithm (a cluster algorithm for graphs).

Several bioinformatics analyses tackled the problem of ceRNA networks in the molecular pathogenesis of glioma [23,24,25]; however, they did not examine the expression of ceRNA networks in glioma cells treated with hypoxia. In this work, we aimed to deepen the understanding of glioma complex molecular pathology, with an emphasis on hypoxia-induced GBM cells, by applying several bioinformatics tools to publicly deposited data from high-throughput experiments [26,27,28]. By the construction of putative GBM ceRNA networks, we explored the molecular context of the hsa_circ_0001081/miR-26b-5p axis and the role of its downstream targets in glioma pathogenesis and GBM molecular subtypes. For this purpose, we combined various R packages, bioinformatics databases, and online tools. Figure 1 shows the general workflow of the bioinformatics analysis.

## 2. Materials and Methods

### 2.1. Differential Expression Analysis

To obtain hypoxia-induced differentially expressed mRNAs (genes; hiDEGs), miRNAs (hiDEMs), and differentially expressed circRNAs (DECs), we used publicly available data deposited in the Gene Expression Omnibus. We extracted raw data from the following records: GSE245800, which contains microarray data from the Illumina HumanHT-12 V4.0 expression beadchip (deposited as a part of paper by de Oliveira et al. [29]); GSE142719, which contains miRNA-seq data from the Illumina HiSeq 2500 (deposited as a part of a paper by Wang et al. [30]) and GSE146463, which contains microarray data from the Arraystar Human CircRNA microarray V2 (deposited as a part of a paper by Bronisz et al. [31]). We uploaded corresponding metadata using the GEOquery package. For data cleaning, we used several R packages, including dplyr. For GSE142719 (miRNA-seq), we extracted high-throughput data considering only wild-type cell samples (three biological replicates of the U-87 MG sample in hypoxia vs. three biological replicates of the U-87 MG sample in normoxia); for GSE245800 (microarray) we utilized all deposited samples (three biological replicates of the U-87 MG sample in hypoxia vs. three biological replicates of the U-87 MG sample in normoxia) and for GSE146463 (circ-microarray) we utilized all deposited samples as well (eight patient-derived glioblastoma cell samples vs. three samples of neural progenitor cells (NPC); note that those samples were not treated with hypoxic conditions). For the differential expression analysis of microarray data (GSE245800 and GSE146463), we used the limma package, and for the differential expression analysis of miRNA-seq data (GSE142719), we used the deseq2 package. For the conversion of Arraystar circRNA IDs to circBase IDs and then to circBank IDs, we used the circPrimer2.0 website “Convert ID” tool (https://www.bio-inf.cn/, accessed on 23 June 2024) and circBank Database (http://www.circbank.cn/, accessed on 13 August 2024), respectively. To visualize the results of the differential expression analysis, we utilized the ggrepel and ggplot2 packages.

### 2.2. The Protein–Protein Interaction (PPI) Network Construction and GO Enrichment Analysis

For the PPI network construction, we uploaded hypoxia-induced differentially expressed genes with their corresponding log_2_FC values into Cytoscape 3.10.1 (https://cytoscape.org/, accessed on 13 August 2024). We took into consideration genes with |log_2_FC| > 1 and *p.adjusted* < 0.05. Using the STRING (Cytoscape app stringApp v2.1.1: https://apps.cytoscape.org/apps/stringapp, accessed on 13 August 2024), we constructed the PPI network based on physical interactions with a default 0.4 confidence score; we used Entrez Gene IDs from the GSE245800 dataset as query terms. For MCL clustering of the PPI network, we used the Cystoscape app clusterMaker2 v2.3.4 (https://apps.cytoscape.org/apps/clustermaker2, accessed on 13 August 2024). We utilized the Cytoscape app Legend Creator v1.1.7 (https://apps.cytoscape.org/apps/legendcreator, accessed on 13 August 2024) to add legends corresponding to the log_2_FC values from the differential expression analysis.

For the GO enrichment analysis of different sets of hiDEGs, we utilized the clusterProfiler, org.Hs.eg.db, and AnnotationDbi packages; we queried the analysis with the gene symbols obtained from the GSE245800 dataset.

### 2.3. The ceRNA Network Construction

To elucidate the interactions among differentially expressed circRNAs (DECs), hypoxia-induced differentially expressed miRNAs (hiDEMs), and hypoxia-induced differentially expressed mRNAs (hiDEGs), we integrated the results from differential expression analyses with miRNA/mRNA and circRNA/miRNA interactions archived in miRTarBase (https://mirtarbase.cuhk.edu.cn/~miRTarBase/miRTarBase_2022/php/index.php, accessed on 17 June 2024) and circBank (http://www.circbank.cn/, accessed on 19 June 2024), respectively. This integration was performed by utilizing the dplyr package. First, we selected hiDEGs with |log_2_FC| > 1 and *p.adjusted* < 0.05 and combined them with their relevant miRNAs obtained from miRTarBase. Then, we intersected the miRNAs/hiDEGs pairs with hiDEMs with |log_2_FC| > 0.5 and *p.adjusted* < 0.05, which resulted in a panel of hiDEMs/hiDEGs interactions. Next, by the selection of DECs with |log_2_FC| > 2 and *p.adjusted* < 0.05 and combining them with circRNA/miRNA pairs from circBase, we obtained DECs/miRNAs pairs. Finally, we intersected pairs of DECs/miRNAs and hiDEMs/hiDEGs, which resulted in putative DECs/hiDEMs/hiDEGs networks. We constructed two types of ceRNA networks: one which consisted of upregulated hiDEGs/downregulated hiDEMs/upregulated DECs (up-hiDEGs/down-hiDEMs/upDECs) and a second which consisted of downregulated hiDEGs/upregulated hiDEMs/upregulated DECs (down-hiDEGs/up-hiDEMs/upDECs). Note that the GSE245800 dataset does not contain hypoxia-induced cells, and thus, constructed ceRNA networks cannot be considered hypoxia-specific; this notion can only be applied to the mRNA/miRNA component of this axis (hiDEGs/hiDEMs). For visualization of the interactions in the ceRNA networks (Sankey diagrams), we utilized the highcharter package.

### 2.4. Glioma Bio Discovery Portal Molecular Subtype and Survival Analyses

We used the Glioma Bio Discovery Portal (Glioma-BioDP; https://glioma-biodp.nci.nih.gov/, accessed on 13 August 2024) to extract plots for GBM molecular subtype analysis. The GBM gene expression data utilized by the Glioma-BioDP comes from The Cancer Genome Atlas-glioblastoma cohort (TCGA-GBM cohort), and the GBM molecular subgroups in our work were based on the microarray data collected from three different platforms (Three platform aggregates: Affymetrix, Agilent, Exon arrays) and subtyped according to Verhaak et al. [32]. Differences in expression levels of the examined genes between pairs of each subtype were compared by *t*-test; we assumed that a *p* < 0.05 means that the difference is statistically significant.

For survival analysis, we obtained data from the TCGA-GBM cohort deposited in the GDC Data Portal (https://portal.gdc.cancer.gov/, accessed on 19 June 2024) using the TCGAbiolinks package. A Kaplan–Meier analysis and log-rank test were conducted using the survminer and survival packages. We assumed that a log-rank *p* < 0.05 was statistically significant.

### 2.5. Package Utilization, Coding, and Generated Data

The R packages used in the study are archived in The Comprehensive R Archive Network (CRAN: https://cran.r-project.org/, accessed on 13 August 2024) and Bioconductor (https://www.bioconductor.org/, accessed on 13 August 2024). All R packages were utilized in RStudio 2024.04.2 (Posit Software, PBC, Boston, MA, USA). We deposited the R code and raw results from the study in Zenodo (https://zenodo.org/, accessed on 13 August 2024) under DOI 10.5281/zenodo.13770443; the record contains raw .csv and .xlsx files obtained during the course of the study, with the names corresponding to particular steps of the bioinformatics workflow (see Figure 1).

## 3. Results

### 3.1. Hypoxia-Specific Differentially Expressed Genes in U-87 MG Are Associated with Extracellular Collagen Biosynthesis

**Figure 2:**
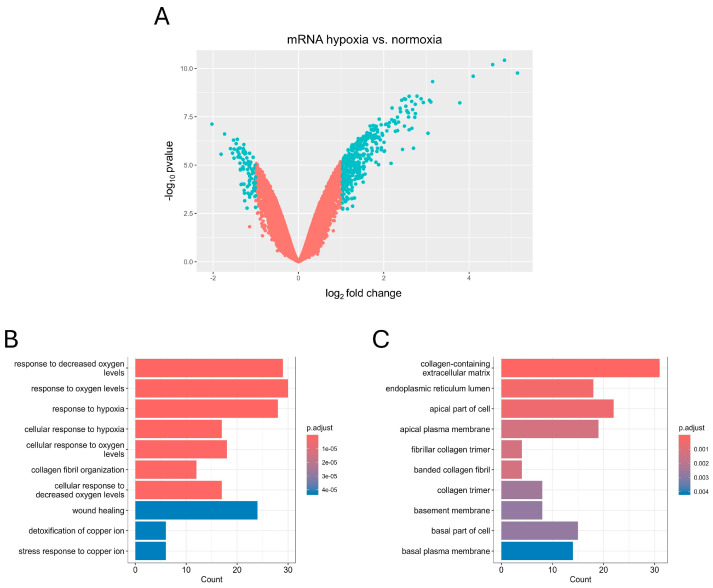
Differential expression (DE) analysis of genes in the hypoxia-induced U-87 MG cell line; (**A**) volcano plot for hypoxia-specific differentially expressed genes (hiDEGs) in U-87 MG cell line, threshold for significant genes (in blue): |log_2_FC| > 1 and *p.adjusted* < 0.05; gene ontology enrichment analysis of biological processes (**B**) and cellular components (**C**) for hypoxia-specific differentially expressed genes (hiDEGs) in U-87 MG cell line.

**Figure 3:**
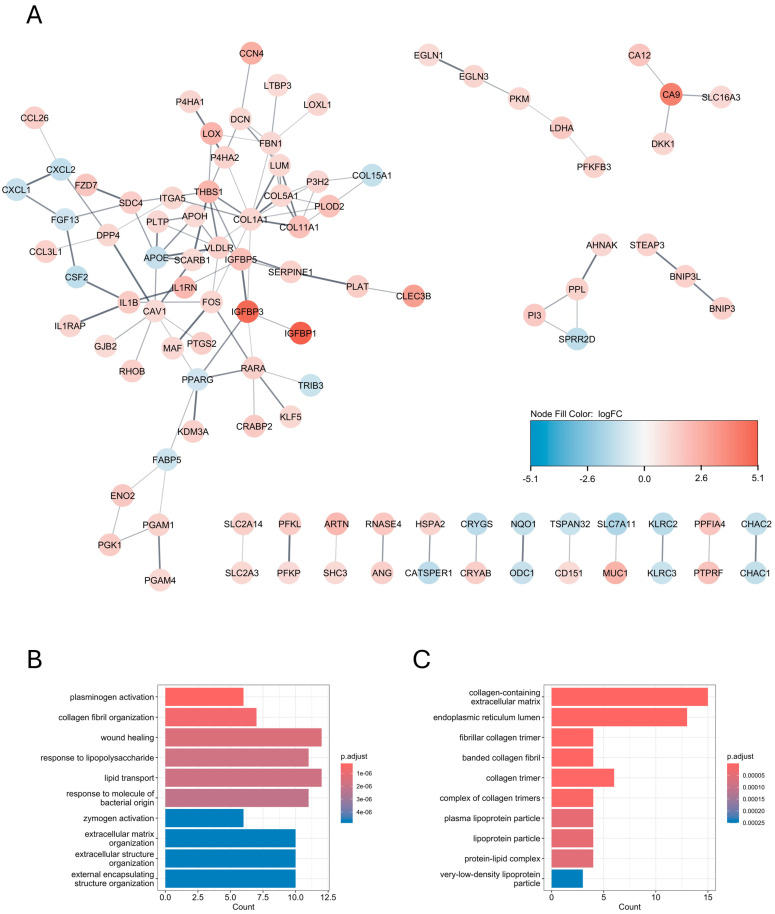
Protein–protein interaction (PPI) clusters for hypoxic genes in U-87 cells; (**A**) PPI network of hypoxia-specific differentially expressed genes (hiDEGs) in U-87 MG cell line with the biggest cluster composed of 57 proteins, where node color corresponds to log_2_FC values from differential expression (DE) analysis, singletons are omitted; gene ontology enrichment analysis of biological processes (**B**) and cellular components (**C**) for the biggest cluster of PPI network in hypoxia-induced U-87 MG cells.

To conduct the differential expression analysis of hypoxia-specific genes, we utilized the limma package on microarray data of three biological replicates of U-87 MG cells treated with hypoxia and three biological replicates of U-87 MG cells cultured in normoxic conditions, deposited in GEO under the accession number GSE245800 [29]. For |log_2_FC| > 1 and *p.adjusted* < 0.05, there were 369 upregulated and 90 downregulated hypoxia-induced differentially expressed genes (hiDEGs) (Figure 2A). The Gene Ontology enrichment analysis for hiDEGs showed that most enriched biological processes mainly correspond to the oxygen level and hypoxia, which is not surprising when considering the design of the study (Figure 2B). In turn, the GO enrichment analysis for the cellular components showed several highly enriched terms corresponding to collagen maintenance, with the term “collagen-containing extracellular matrix” being the most enriched (Figure 2C). To further elucidate the role of hypoxia-associated protein interactions, we constructed the PPI interaction network of hiDEGs with physical interactions subnetworks, and the default confidence score equaled 0.4 using stringApp v2.1.1 (Figure 3A). The GO enrichment analysis for the biological processes and cellular components of the biggest cluster composed of 57 proteins showed highly enriched terms corresponding to collagen biosynthesis, with the “collagen-containing extracellular matrix” being the most enriched cellular component term (Figure 3B,C).

### 3.2. Hypoxia Affects Small Fraction of miRNAs with Several Potential Interactions with Protein-Coding Transcripts in U-87 MG Cells

**Figure 4:**
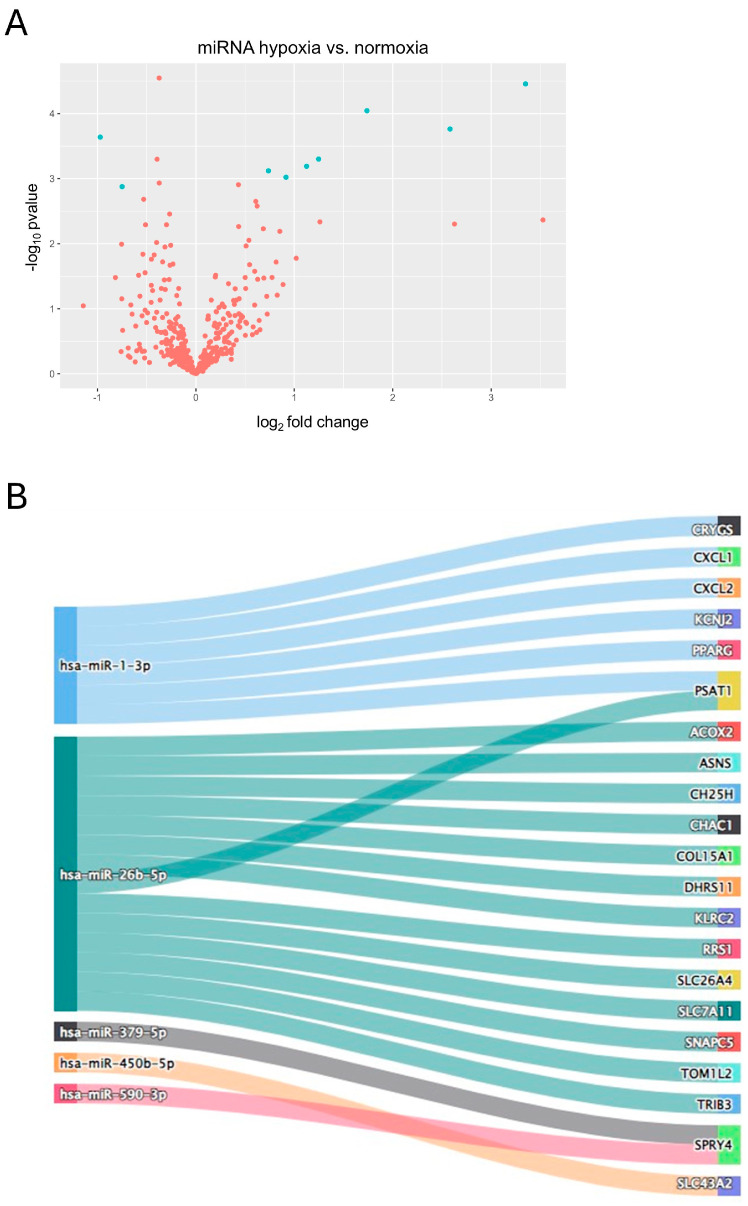
Differential expression analysis of miRNA in hypoxia-induced U-87 MG cell line; (**A**) volcano plot for hypoxia-specific differentially expressed miRNAs (hiDEMs) in U-87 MG cell line, threshold for significant miRNAs (in blue): |log_2_FC| > 0.5 and *p.adjusted* < 0.05; (**B**) Sankey diagram for hypoxia-specific upregulated miRNAs (up-hiDEMs) and their downstream competing endogenous hypoxia-specific downregulated targets (down-hiDEGs) in hypoxia-induced U-87 MG cell line.

For the differential expression analysis of hypoxia-induced miRNAs (hiDEMs), we used high-throughput sequencing data of three biological replicates of the U-87 MG cells treated with hypoxia vs. three biological replicates of the U-87 MG cells cultured in normoxia. These data are available in the GEO under accession number GSE142719 [31]. Deseq2 showed that for |log_2_FC| > 0.5 and *p.adjusted* < 0.05, there were only seven upregulated and two downregulated miRNAs (Figure 4A). Using data from miRTarbase, we intersected hiDEMs with hiDEGs into potential interaction pairs. The Sankey diagram shows constructed interactions of the upregulated hiDEMs (up-hiDEMs) and downregulated hiDEGs (down-hiDEGs) in U-87 MG cells, with miR-26b-5p having the biggest number of potential interactions (Figure 4B).

### 3.3. hsa_circ_0001081/miR-26b-5p May Affect Collagen-Associated Pathways through COL15A1 and TRIB3

**Figure 5:**
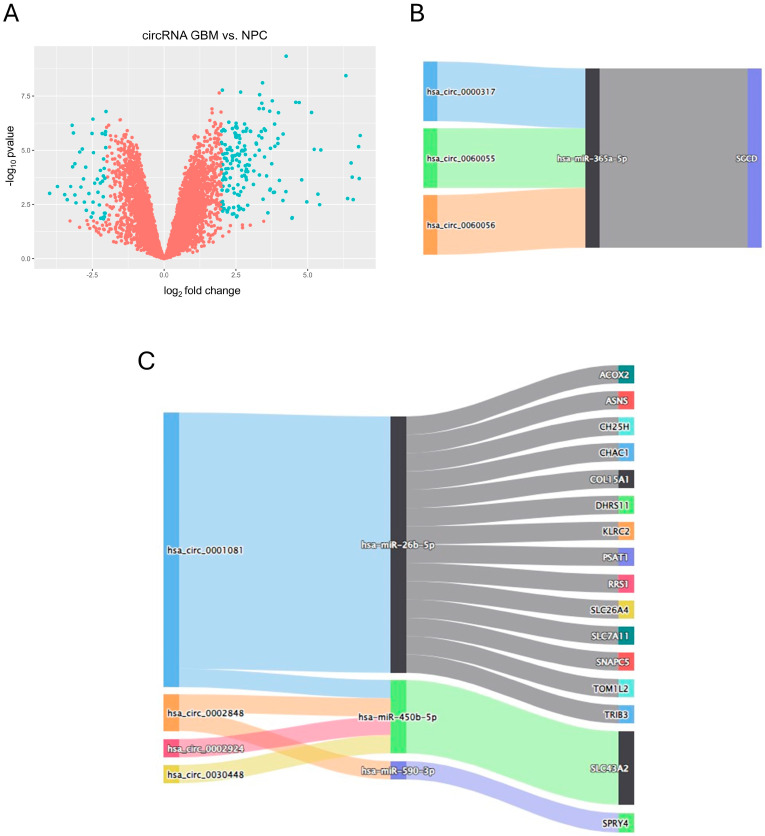
Differential expression (DE) analysis of circRNA in patient-derived GBM cells vs. neural progenitor cells (NPC) and putative GBM-specific ceRNA network construction; (**A**) volcano plot for differentially expressed circRNAs (DECs) in GBM patient-derived cells as compared to neural progenitor cells (NPC), the threshold for significant circRNA (in blue): |log_2_FC| > 2 and *p.adjusted* < 0.05; (**B**) Sankey diagram of putative GBM ceRNA network of 3 upregulated differentially expressed circRNAs (upDECs), hypoxia-specific downregulated miR-365a-5p, and hypoxia-specific upregulated *SGCD*; (**C**) Sankey diagram of putative GBM ceRNA network of downregulated differentially expressed circRNAs (downDECs), hypoxia-specific upregulated miRNAs (up-hiDEMs), and hypoxia-specific downregulated genes (down-hiDEGs).

**Figure 6:**
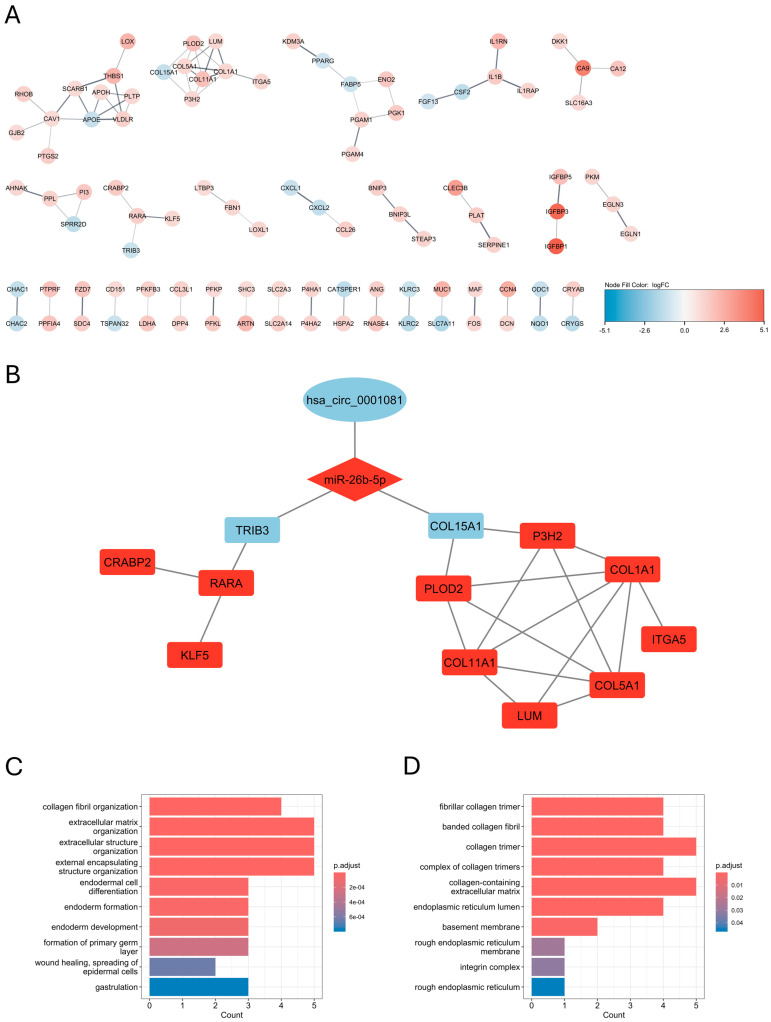
Effect of the hsa_circ_0001081/miR-26b-5p axis on PPI network subclusters; (**A**) MCL clustering of the PPI network obtained from hypoxia-specific differentially expressed genes (DEGs) in U-87 MG cell line, granularity parameter = 3, node color corresponds to log_2_FC values from differential expression (DE) analysis of GSE245800 dataset, singletons are omitted; (**B**) putative GBM ceRNA network of hsa_circ_0001081/miR-26b-5p/*COL15A1*/*TRIB3* and two downstream subclusters obtained from MCL clustering of PPI network; gene ontology enrichment analysis of biological processes (**C**) and cellular components (**D**) for COL15A1-containing subcluster of PPI network.

To obtain a list of DECs, we conducted differential expression analysis using limma on the microarray data deposited in the GEO under the accession number GSE146463 [30]. Although this record contains data from eight samples of patient-derived cells and three samples of neural progenitor cells (NPC), we decided to intersect differentially expressed circRNA in glioblastoma cells vs. NPC with hypoxia-specific networks to select potentially valuable prospects among the circular transcripts for potential further research. The differential expression analysis showed 174 upregulated and 48 downregulated circRNAs (Figure 5A). By combining the circRNA/miRNA interactions deposited in circBank with DECs and then hiDEMs/hiDEGs pairs, we obtained final DECs/hiDEMs/hiDEGs interaction networks. According to the ceRNA hypothesis, we constructed two types of networks: first, in which upregulated circRNAs sponge miRNAs to rescue their inhibitory effect on downstream mRNAs (upDECs/down-hiDEM/up-hiDEG) (Figure 5B); second, in which upregulated miRNA inhibits one of the ceRNAs and its activity is not impeded due to the downregulation of another competing molecule in the axis (downDECs/up-hiDEMs/down-hiDEGs) (Figure 5C). The first network revealed three circRNAs with potential impact on the miR-365a-5p/*SGCD* axis; however, the protein-coding component of this ceRNA network held no interactions in the constructed PPI network (as SGCD was a singleton, it is omitted in Figure 3A). On the other hand, two protein-coding genes in the second network, *COL15A1* and *TRIB3*, were present in the biggest collagen-enriched cluster in the PPI network analysis (Figure 3A). Both *COL15A1* and *TRIB3* were affected by the hsa_circ_0001081/hsa-miR-26b-5p axis (Figure 5C). MCL clustering with granularity parameter 3 revealed seven subclusters, which included more than three proteins each (Figure 6A). There was one subcluster, which included COL15A1 and seven other proteins (COL1A1, COL5A1, COL11A1, ITGA5, P3H2 (LEPREL1), LUM, and PLOD2), and one subcluster, which included TRIB3 and three other proteins (RARA, CRABP2, and KLF5). Figure 6B shows the whole putative ceRNA network of the hsa_circ_0001081/hsa-miR-26b-5p/*COL15A1*/*TRIB3* axes with their downstream subclusters. For the COL15A1-including subcluster, the GO enrichment analysis for the biological processes and cellular components showed several highly enriched terms associated with extracellular matrix collagen biosynthesis (Figure 6C,D). Thus, we concluded that hsa_circ_0001081/hsa-miR-26b-5p may affect collagen biosynthesis and maintenance through *TRIB3* and especially through *COL15A1* at the transcriptomic level.

### 3.4. hsa_circ_0001081/miR-26b-5p/COL15A1-Affected Collagen-Enriched Subcluster Can Help Distinguish Mesenchymal Glioma Subtype from Other Subtypes

The conducted analyses aimed to investigate whether the expression levels of hypoxia- and collagen-associated genes reflect the transcriptomic profiles that are characteristic of the mesenchymal phenotype in GBM. For this purpose, subtype expression analysis was performed on the TCGA-GBM cohort using the Glioma Bio Discovery Portal. Additionally, to assess the potential prognostic value of the genes influenced by the hsa_circ_0001081/miR-26b-5p axis, a survival analysis was conducted on the TCGA-GBM cohort, which was obtained via the GDC portal with the TCGAbiolinks package.

It is important to note that the molecular subtyping of GBM has undergone significant revision in recent years. Specifically, the “neural subtype” is no longer recognized as a distinct entity within GBM. It has been demonstrated that cases previously classified as “neural glioma” predominantly consist of non-cancerous neural cells located at the margins of surgical resections [11,33]. Consequently, the “neural subtype” should be excluded from the subtype expression analysis to ensure the accuracy and relevance of the findings.

**Figure 7:**
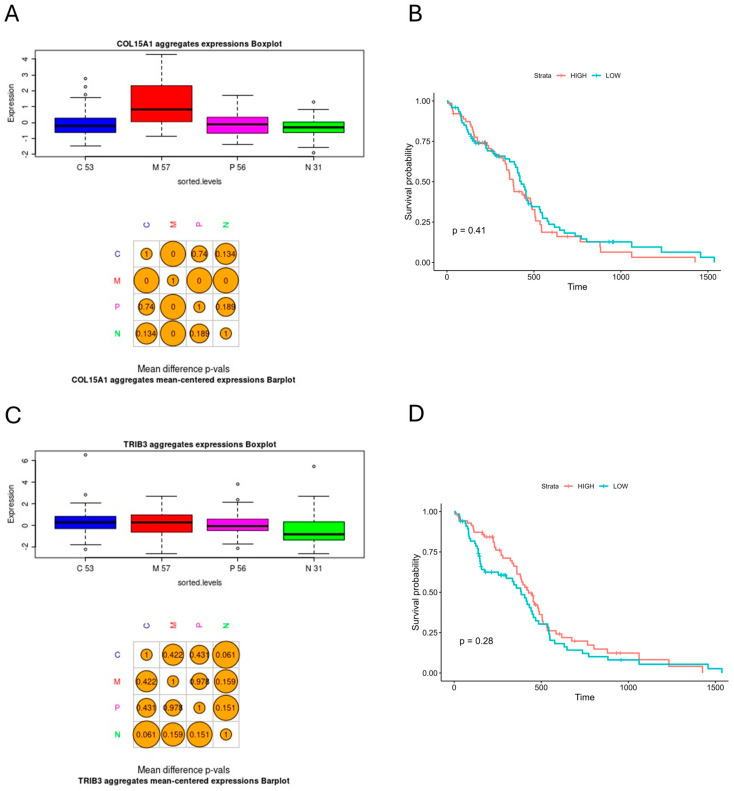
Subtype expression and survival analyses of the TCGA-GBM cohort for (**A**,**B**) *COL15A1*; (**C**,**D**) *TRIB3*; barplottted t-test where *p* < 0.05 is statistically significant, and a log-rank *p* < 0.05 is statistically significant; C—classical GBM subtype; M—mesenchymal GBM subtype; P—proneural GBM subtype; N—neural GBM subtype (note that neural subtype is no longer recognized by newer classification); numbers under box plots refer to the size of the individual subgroups.

Figure 7 shows the results for *COL15A1* and *TRIB3*, two genes that were targeted directly by the hsa_circ_0001081/miR-26b-5p axis. For *TRIB3*, there were no significant differences in its expression level between molecular subtypes; interestingly, *COL15A1* exerted a significantly elevated expression level in the mesenchymal subgroup as compared to both proneural and classical subgroups, although in hypoxia-induced U-87 MG cells, it exhibited decreased expression. The Kaplan–Meier analysis with a log-rank test revealed no statistically significant differences in patients’ survival regarding the expression levels of *COL15A1* and *TRIB3* in the examined cohort.

**Figure 8:**
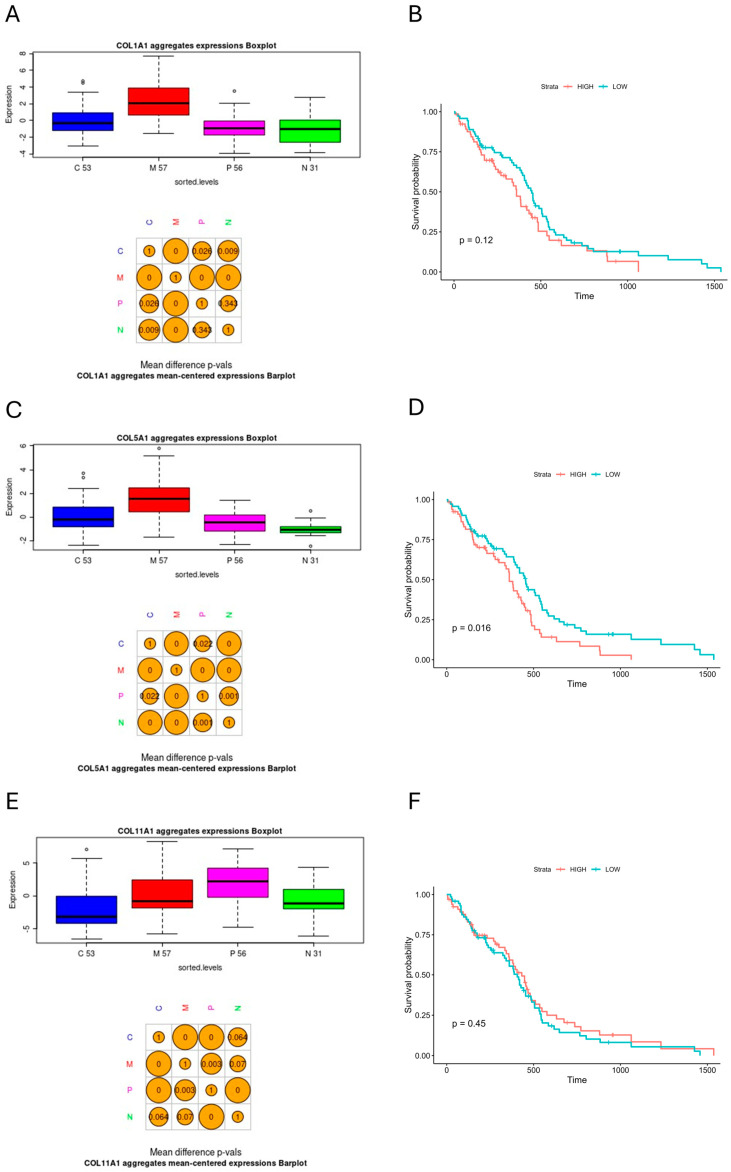

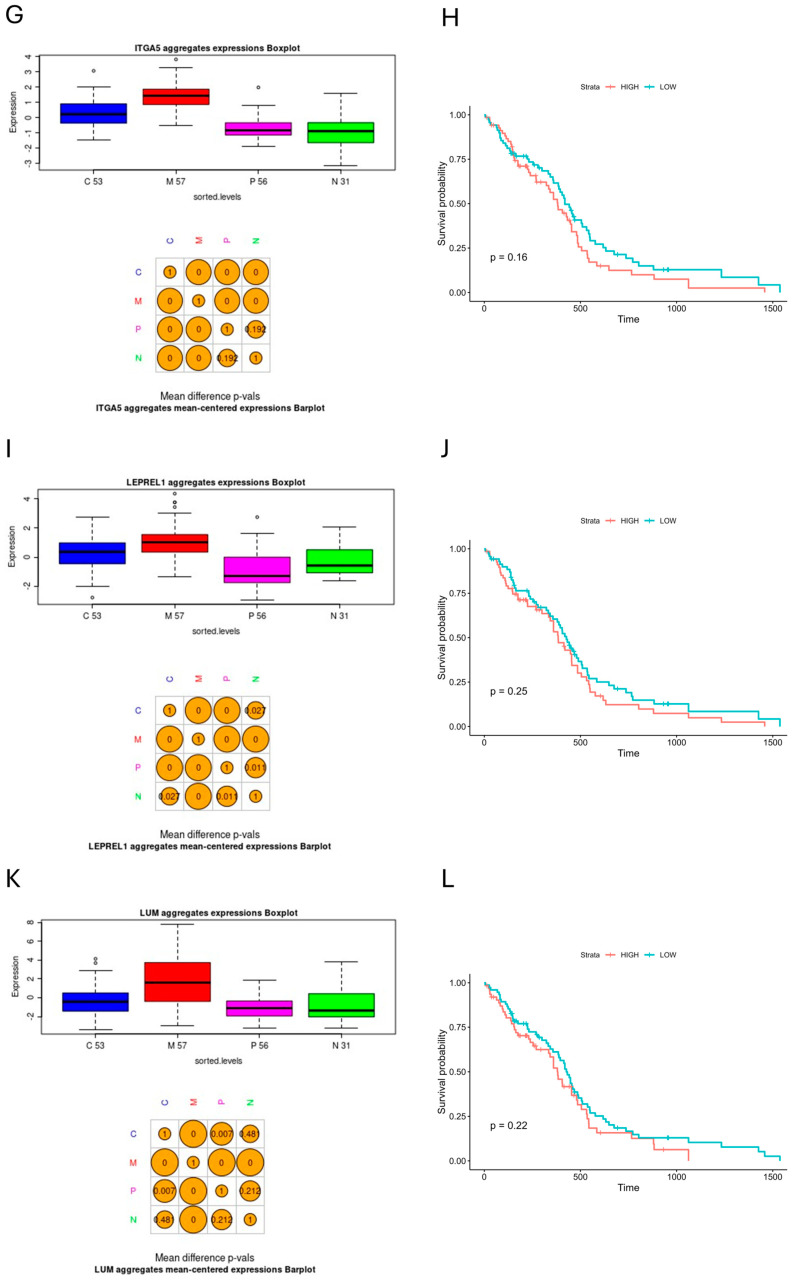

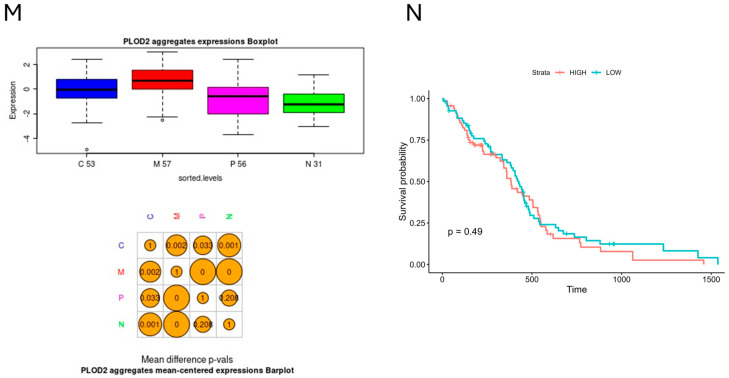
Subtype expression and survival analyses of TCGA-GBM cohort for genes included in COL15A1-affected subcluster from PPI network; (**A**,**B**) *COL1A1*, (**C**,**D**) *COL5A1*, (**E**,**F**) *COL11A1*, (**G**,**H**) *ITGA5*, (**I**,**J**) *LEPREL1* (*P3H2*), (**K**,**L**) *LUM*, and (**M**,**N**) *PLOD2*; barplottted *t*-test *p* < 0.05 is statistically significant, log-rank *p* < 0.05 is statistically significant. C—classical GBM subtype; M—mesenchymal GBM subtype; P—proneural GBM subtype; N—neural GBM subtype (note that neural subtype is no longer recognized by newer classification); numbers under box plots refer to the size of the individual subgroups.

For further analysis, we subjected genes that encode the proteins included in the collagen-enriched subcluster from the PPI network (Figure 6B–D). *COL1A1*, *COL5A1*, *ITGA5*, *LEPREL1* (*P3H2*), *LUM*, and *PLOD2* exhibited significantly higher expression levels in the mesenchymal subgroup as compared to other subgroups (Figure 8A,C,G,I,K,M). *COL11A1* was the only gene with the highest expression in the proneural subtype compared to others, but its expression in the mesenchymal subtype was still significantly higher than in the classical subtype (Figure 8E). The Kaplan–Meier analysis with a log-rank test showed that among the examined genes, high expression of only *COL5A1* is a significant predictor of poor patient survival (*p* = 0.016, Figure 8D); however, high expression levels of *COL1A1*, *ITGA5*, *LEPREL1* (*P3H2*), and *LUM* exhibited non-significant trends in worsening patient survival as well (Figure 8B,H,J,L). These tendencies were unclear in the case of *COL11A1* and *PLOD2* (Figure 8F,N).

Thus, we concluded that the GBM hsa_circ_0001081/miR-26b-5p/*COL15A1*-affected subcluster of collagen-enriched genes can help distinguish the mesenchymal glioma molecular subtype from other molecular subtypes. High expression levels of *COL5A1* may predict poor prognosis in GBM patients.

## 4. Discussion

In this study, we sought to deepen the understanding of GBM molecular interactions under hypoxic conditions by constructing hypoxia-specific miRNA/mRNA networks using U-87 MG cell line data. Our analysis identified miR-26b-5p as a key regulator, targeting a significant number of hypoxia-downregulated genes in this cell line. Further investigation revealed a potential circRNA/miRNA/mRNA axis involving hsa_circ_0001081, miR-26b-5p, *COL15A1*, and *TRIB3*, suggesting a complex interplay between these molecules in the hypoxic GBM microenvironment.

To date, several reports assessed miR-26b-5p expression levels in various malignancies, including lymphoma, breast, and gastric cancer [34,35,36]. These studies are consistent in their conclusions regarding the tumor-suppressing activity of miR-26b-5p in carcinogenesis, although some types of hematological malignancies are not in line with that statement [37]. In terms of glial tumors, an article by Wu et al. highlighted the role of miR-26b in glioma by comparing its expression level across different glioma grades, which showed decreasing levels of this microRNA along with an increasing WHO grading of glioma patients [38]. As hypoxic states in GBM are associated with aggressive behavior, especially in the more invasive mesenchymal phenotype, it is tempting to connect hypoxia-induced molecules with those exhibiting oncogenic properties [11,12,13]. However, our analysis showed that miR-26b-5p was significantly upregulated in U-87 MG cells under hypoxic conditions, which does not mirror this simple assumption. Construction of miRNA-mRNA networks based on the interactions obtained from miRTarBase showed 14 mRNAs potentially targeting miR-26b-5p (Figure 4B). Even if miR-26b-5p plays a general role as a tumor suppressor in GBM, it is possible that, in hypoxic conditions, its downregulated competing endogenous RNAs cannot exert their inhibitory activation, which leads to its elevated expression level. Interestingly, Dai et al. showed that hypoxia can induce macrophages to secrete exosomal miR-26b-5p, which, when introduced into keloid fibroblasts, may promote EMT and fibrosis [39].

On the other hand, the PPI network analysis, along with the GO enrichment analysis of the hypoxia-specific genes in U87-MG cells, showed that COL18A1 and TRIB3, which may be negatively affected by miR-26b-5p at the transcriptomic level, are part of a large protein cluster by which they share a collagen-associated crosstalk (Figure 3). This effect may be potentiated by the downregulation of hsa_circ_0001081, which may interact with downstream miR-26b-5p targets in a ceRNA hypothesis manner (Figure 5C). The MCL clustering and GO enrichment analyses revealed certain hypoxia-specific subcluster as being particularly enriched in collagen-associated terms, composed of COL15A1, COL1A1, COL5A1, COL11A1, ITGA5, LEPREL1 (P3H2), LUM, and PLOD2 (Figure 6A,C,D). We further subjected this panel to GBM subtype expression analysis in the Glioma Bio Discovery Portal. For seven hypoxia-upregulated genes belonging to that panel, six exhibited a significantly higher expression in the GBM mesenchymal subtype as compared to the classical and proneural subtypes, and one exerted a significantly higher expression level in the GBM proneural subtype as compared to the other subtypes but was still highly expressed in the mesenchymal group as compared to the classical group (Figure 8). Nonetheless, this tendency of hypoxia-upregulated genes being at least to some degree a reflection of the GBM mesenchymal phenotype is another confirmation of the already mentioned notion, which links mesenchymal GBM niches with hypoxic conditions [11,12,13]. In the context of our analyses, it rather highlights the association between collagen-associated processes and GBM mesenchymal transformation.

In turn, COL15A1 was the only molecule of the collagen-enriched subcluster, which does not mirror the assumption that U-87 MG cells treated with hypoxia can be easily interpreted as mesenchymal GBM phenotype (Figure 7A). Although the differential expression analysis of the GSE245800 dataset showed that *COL15A1* was downregulated in hypoxic conditions, in the TCGA-GBM cohort, it exerted significantly higher expression in the mesenchymal subgroups as compared to both the proneural and classical subtypes, similarly to other *COL* family members in the shared subcluster. Interestingly, Lambertini et al. showed that Hif-1α increases *COL15A1* expression by binding to its promoter in a hypoxic mesenchymal stromal cells model [40]. This suggests that *COL15A1* may have a unique role in U-87 MG cells, possibly acting as a “lonely island” among collagen-related genes that are upregulated under similar conditions. Apparently, the role of *COL15A1* in cancer seems to be a complex problem; it exhibited elevated levels of expression in various chemoresistant ovarian cancer cell lines [41], but its inhibition promoted an aggressive phenotype in the hepatoblastoma cell line [42]. These reports, along with the results of our analysis, highlight the need for further research of *COL15A1* regarding glial tumors, as well.

Another ceRNA putatively targeted by miR-26b-5p is *TRIB3*—a ferroptosis-related gene whose high expression has been associated with tumor progression [43,44,45]. Wu et al. reported its association with extracellular matrix collagen deposition by means of functional enrichment analysis [44]. In a study by Tang et al., it was shown to possess increased expression and a positive effect on migration in U-87 MG cells [46]. In fact, this is logical in the context of our analysis, as the hypoxia-upregulated U87-MG-specific miR-26b-5p (as it was mentioned a “general suppressor”) can potentially inhibit *TRIB3* activity (“glioma oncogene”) in a ceRNA manner.

Considering the above points, it seems that the exact effect of miR-26b-5p on the extracellular matrix in hypoxic U-87 MG cells is difficult to assess. Even if it decreases *TRIB3* expression (which presumably encodes “collagen inducing protein”), it may still interact with *COL15A1*, which is a part of the most collagen-enriched PPI subnetwork and interacts with other collagen-associated proteins (Figure 6). Therefore, the extent to which this collagen-associated subcluster may be influenced by miR-26b-5p needs to be further elucidated. The question should be rather stated: What exact modulation on collagen-associated proteins does miR-26b-5p/*COL15A1*/*TRIB3* axes exert in U-87 MG cells in terms of their particular influence on specific cellular collagens expression, and how (if at all) do those traits become translated into the extracellular matrix composition in vivo?

Additionally, while our analyses indicate a potential role for hsa_circ_0001081 in the miR-26b-5p/*COL15A1*/*TRIB3* axis, the lack of in-depth studies on this circRNA in GBM underscores the need for further research. Given that *DCAF17*, the host gene of hsa_circ_0001081, is linked to neurodegenerative diseases [47], exploring its circular variant’s role in GBM pathogenesis could provide novel and valuable insights in the field of glioma research.

It is important to acknowledge the limitations of this study. Our findings are based solely on bioinformatics analyses, requiring experimental validation to confirm the hypothesized regulatory axes. Moreover, the analyzed datasets were derived from a limited number of cell line samples, and the circRNA data were not specific to hypoxia but rather compared patient-derived cells with normal counterparts. Future studies should focus on validating these axes in larger, uniformly treated samples to establish their relevance in GBM pathology.

Our findings challenge the straightforward association of hypoxia-induced molecules with oncogenic properties and highlight the potential context-dependent role of miR-26b-5p in GBM. We propose that in U87-MG cells, miR-26b-5p may be a hypoxia-induced regulator which, by targeting downstream *COL15A1* and *TRIB3*, may affect extracellular collagen deposition. Additionally, circ_0001081 may be a potential regulator of those axes in a ceRNA hypothesis manner. Several collagen-associated genes, including *COL1A1*, *COL5A1*, *ITGA5*, *P3H2*, *LUM,* and *PLOD2*, may help distinguish between mesenchymal and other molecular subtypes of GBM. High expression levels of *COL5A1* may be a poor prognostic predictor for GBM patients. Further laboratory studies are needed to support these statements.

## Data Availability

The data presented in this study are available in the Gene Expression Omnibus at https://www.ncbi.nlm.nih.gov/geo/, [accessed on 13 August 2024], reference numbers GSE245800, GSE142719, and GSE146463. These data were derived from the following resources available in the public domain: https://www.ncbi.nlm.nih.gov/geo/query/acc.cgi?acc=GSE245800 [accessed on 16 June 2024]; https://www.ncbi.nlm.nih.gov/geo/query/acc.cgi?acc=GSE142719 [accessed on 8 June 2024]; https://www.ncbi.nlm.nih.gov/geo/query/acc.cgi?acc=GSE146463 [accessed on 23 June 2024]; The original data presented in the study are openly available in Zenodo at DOI 10.5281/zenodo.13770443.
